# Effects of Thermal Exposure Temperature on Room-Temperature Tensile Properties of Ti65 Alloy

**DOI:** 10.3390/ma17174424

**Published:** 2024-09-09

**Authors:** Yuan-Chen Wang, Jian-Yang Liu, Jian-Rong Liu, Wen-Yuan Li, Bin Zhang, Guang-Ping Zhang

**Affiliations:** 1 Institute of Metal Research, Chinese Academy of Sciences, Shenyang 110016, China; ychwang19b@imr.ac.cn (Y.-C.W.); wyli@imr.ac.cn (W.-Y.L.); 2 Key Laboratory for Anisotropy and Texture of Materials (Ministry of Education), School of Materials Science and Engineering, Northeastern University, Shenyang 110819, China; 18732816077@163.com (J.-Y.L.); zhangb@atm.neu.edu.cn (B.Z.)

**Keywords:** Ti65 alloy, thermal exposure, tensile property, oxide layer, precipitate, silicide

## Abstract

As a critical material for high-temperature components of aero-engines, the mechanical properties of Ti65 alloy, subjected to high-temperature and long-term thermal exposure, directly affect its service safety. The room-temperature tensile properties of the Ti65 alloy after thermal exposure to temperatures ranging from 450 °C to 650 °C for 100 h were investigated. The results indicate that as the thermal exposure temperature increases, the strength of Ti65 alloy initially increases and then decreases, while ductility exhibits a decreasing trend. The strength of the thermally exposed alloy positively correlates with the size and content of the *α*_2_ phase. The ductility of the thermally exposed alloy is comprehensively influenced by the surface oxidation behavior, *α*_2_ phase, and silicides. After the prolonged thermal exposure, stress concentration at the crack tips within the oxide layer was enhanced with the increased thickness of the surface TiO_2_ oxide layer, leading to premature fracture due to reduced alloy ductility. Furthermore, the *α*_2_ phase in the matrix promotes the planar slip of dislocations, while silicides at the *α*/*β* phase boundaries hinder dislocation motion, causing dislocation pile-ups. Both behaviors facilitate crack nucleation and deteriorate alloy ductility.

## 1. Introduction

High-temperature titanium alloys are extensively utilized in the fabrication of compressor components and casings positioned proximate to the combustor in aviation engines, owing to their advantageous properties, including their exceptional specific strength, robust corrosion resistance, low density, and outstanding high-temperature performance, as reported in [1,2]. In recent years, with the advancement in aerospace technology, more stringent requirements have been put forward for the performance indicators of aviation engines, which makes it particularly important to evaluate and predict the service reliability and life of this kind of material in a high-temperature environment using data-driven methods [3,4,5]. Meanwhile, it is imperative to develop the high-temperature titanium alloys with higher operating temperatures and improved performance [6]. The Ti65 alloy is a near-alpha (*α*) high-temperature titanium alloy strengthened by silicides, designed to operate at temperatures of 600 °C to 650 °C and thus is expected to be used for manufacturing aviation engine rotors, blades, and critical high-temperature structural components [7]. These hot-end components are subjected to long-term exposure to high-temperature atmospheric environments during service, making their thermal stability crucial for ensuring the safety of the components.

Regarding alloy surface stability, during the long-term high-temperature exposure, the alloy will form an oxide layer at the part surface and an oxygen-enriched diffusion layer internally. The comparative analysis of the room-temperature tensile properties of Ti60 alloy with and without the surface oxide layer and oxygen-affected region formed through prolonged thermal exposure at 600~800 °C shows that the tensile strength and ductility of the Ti65 alloy are improved after the removal of the surface oxide layer and oxygen-affected region [8]. Satko et al. [9] predicted the critical strain for crack initiation based on the thickness of the brittle oxygen-enriched layer in the Ti-6424S alloy. They proposed a predictive model for the effect of oxygen-enriched layer thickness on fatigue life. A thicker oxygen-enriched layer leads to a lower critical strain for cracking and consequently reduces the fatigue life of the alloy. Furthermore, regarding the microstructure stability of the alloy, there are changes in the matrix structure and precipitated phases of the alloy during the long-term high-temperature exposure. Zhang et al. [10] found that cyclic thermal stresses during 650 °C/100 h cyclic thermal exposure of the laser-deposited Ti60A alloy promote the penetration diffusion of oxygen, resulting in the formation of more oxygen-rich and coarsened *α* phase layers with lower plasticity, leading to plasticity loss and a significant decrease in mechanical performance. Lunt et al. [11] found that during the thermal exposure, the *α*_2_ ordered phase (Ti_3_Al) in the TG6 alloy gradually accumulates and evolves into a long-range ordered structure. Small *α_2_* phases interact with mobile dislocations during deformation, enhancing the strength of the alloy through the second-phase strengthening. Additionally, as plastic deformation progresses, the *α*_2_ phase promotes the planar slip of mobile dislocations, thus reducing the alloy’s plasticity. Investigations by Madsen et al. [12,13] show that prolonged high-temperature exposure of near-alpha titanium alloys at 593 °C can cause the precipitation of large silicon particles, subsequently reducing fatigue performance of the alloy, tensile ductility, and fracture toughness. The above investigations reveal that both the surface stability and microstructural stability of the alloys subjected to prolonged high-temperature exposure may directly influence their service reliability. It is necessary to evaluate the impact of long-term high-temperature exposure on the mechanical properties and microscopic mechanisms of the Ti65 alloy.

In this study, the Ti65 alloy was subjected to 100 h thermal exposure experiments at temperatures ranging from 450 °C to 650 °C in the air atmosphere. The room-temperature tensile properties of the alloy before and after the thermal exposure were assessed to investigate the influence of different thermal exposure temperatures (*T_e_*s) on the tensile mechanical behavior of the alloy at room temperature. The basic mechanisms for the evolution of the strength and ductility of the alloy with the thermal exposure temperature were elucidated through theoretical analysis.

## 2. Materials and Methods

### 2.1. Materials and Heat Treatment Process

The chemical composition of the Ti65 alloy used in the experiment is listed in Table 1. The alloy was melted in a vacuum arc furnace and forged into discs, and then, samples were cut from them for the experiment. The samples were subjected to a solution treatment through heating at 1014 °C for 2 h followed by water quenching.

### 2.2. Thermal Exposure and Tensile Testing

**Figure 1:**
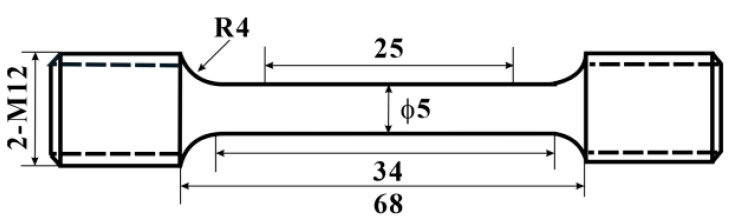
Dimensions of the tensile specimen (GB/T 228.1-2021 [14]).

Rectangular specimens with dimensions of 10 mm × 10 mm × 5 mm and cylindrical tensile specimens with a gauge length of ϕ5 mm × 25 mm (Figure 1) were cut from the solution-treated samples using electrical discharge machining equipment HA320 (SSG, Suzhou, China). Then, the surfaces of two types of specimens were mechanically ground and polished using 100- to 3000-grit sandpaper, followed by ultrasonic cleaning with ethanol for all surface-treated specimens. Finally, all specimens were divided into five groups and subjected to thermal exposure experiments in an air atmosphere in a box-type resistance furnace model SXL-1400C (SIOMM, Shanghai, China). The *T_e_*s for the five groups of specimens were 450 °C, 500 °C, 550 °C, 600 °C, and 650 °C, each maintained for 100 h before being air-cooled. Following these procedures, the rectangular and tensile specimens were respectively used for studying the oxidation layer, microstructure characterization, and tensile properties of the Ti65 alloy after 100 h of thermal exposure at different temperatures.

Tensile property testing of the specimens before and after the thermal exposure was conducted using an Instron 5982 testing machine (Instron, Boston, MA, USA) at room temperature with a strain rate of 3.3 × 10^−4^ s^−1^. Strain measurements of the gauge length section of the specimens were taken using a standard extensometer, with each group of specimens tested three times.

### 2.3. Microstructural Characterization

The grain structure, oxide layer, and fracture morphology of the specimens were observed and analyzed using a field emission scanning electron microscope SUPRA 35 (LEO, Berlin, Germany). For phase composition analysis of the surface oxide layer, an X-ray diffractometer Bruker D8 Discover (Bruker AXS, Karlsruhe, Germany) with a Cu target radiation source was employed, with scanning angles ranging from 20° to 90° at a scanning speed of 4°/min. The precipitated phases of the specimens were characterized and analyzed using a field emission transmission electron microscope FEI Tecnai 20 (FEI, Hillsboro, OR, USA). To protect the oxide layer of the specimens after the thermal exposure, the specimen surface was electroplated with nickel before mechanical grinding and polishing of the cross-section, followed by SEM characterization and analysis of the oxide layer. For a clear observation and characterization of the matrix structure, metallurgical etching treatment was performed on the surface-polished specimen, followed by SEM characterization of the matrix structure. The etchant solution ratio was hydrofluoric acid:nitric acid:water = 2:7:91.

## 3. Results

### 3.1. Microstructure and Precipitated Phases

**Figure 2:**
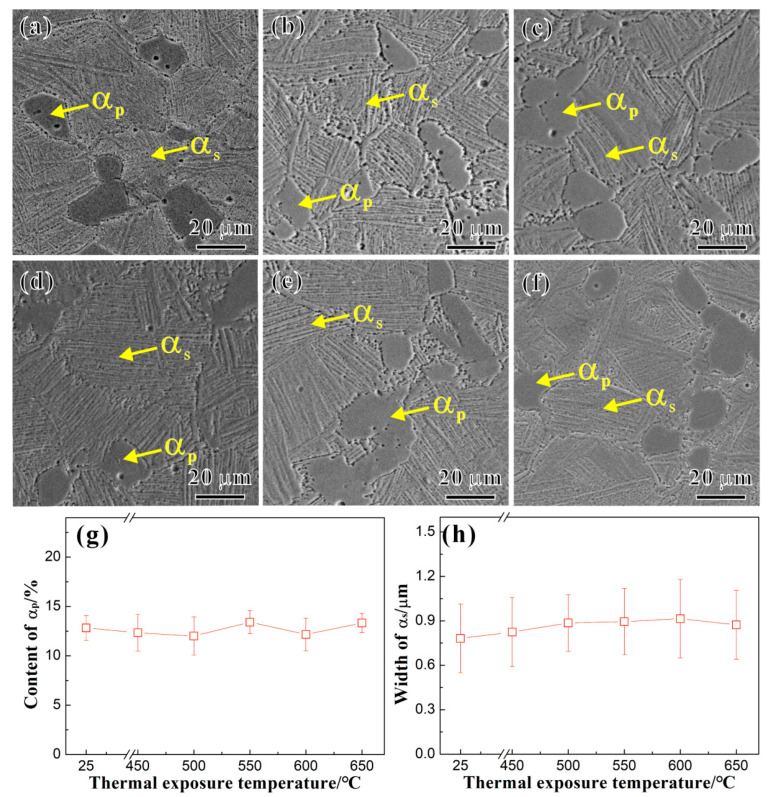
SEM images of Ti65 alloy (**a**) before and after the thermal exposure for 100 h at temperatures of (**b**) 450 °C, (**c**) 500 °C, (**d**) 550 °C, (**e**) 600 °C, and (**f**) 650 °C, respectively, and variation in (**g**) *α_p_* content and (**h**) *α_s_* width with *T_e_* (25 °C represents the unexposed specimen).

Figure 2a shows the SEM image of the matrix structure of the Ti65 alloy before thermal exposure. After the solution treatment, the matrix structure of the Ti65 alloy consists of a dual-phase structure composed of a primary equiaxed *α_p_* phase and transformation structure (*β_trans_*). The primary equiaxed *α_p_* phase is the original *α* phase present after thermal-mechanical processing of the Ti65 alloy, while *β_trans_* is composed of a secondary lath *α* phase (*α_s_*) and residual lath-like phase (*β_r_*) precipitated after solution treatment. Figure 2c–f, respectively, show the SEM images of the matrix structure of the Ti65 alloy after the thermal exposure at 450 °C, 500 °C, 550 °C, 600 °C, and 650 °C for 100 h. The matrix structure of the alloy still consists of a primary equiaxed *α_p_* phase and *β_trans_* after the thermal exposure. Based on numerous SEM images, the statistical results of the area percentage of the primary equiaxed *α_p_* phase and the width of the secondary lath *α_s_* phase in the matrix structure of the Ti65 alloy before and after the thermal exposure are shown in Figure 2g and Figure 2h, respectively. There are no significant changes in the content of the *α_p_* phase and the width of the secondary lath *α_s_* phase with the increase in *T_e_* = 450 °C to 650 °C for 100 h, indicating that thermal exposure at temperatures ranging from 450 °C to 650 °C for 100 h has no significant effect on microstructures of the Ti65 alloy.

**Figure 3:**
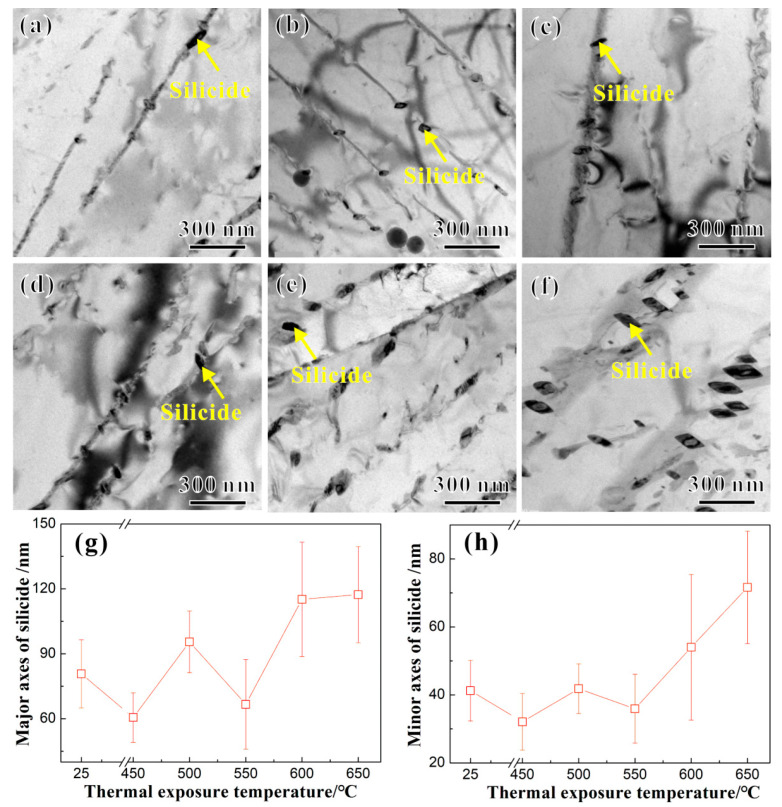
TEM images of silicide in the Ti65 alloy (**a**) before and after the thermal exposure for 100 h at temperatures of (**b**) 450 °C, (**c**) 500 °C, (**d**) 550 °C, (**e**) 600 °C, and (**f**) 650 °C, respectively, as well as the relations between (**g**) major axes and (**h**) minor axes and heat exposure temperatures (25 °C represents the unexposed specimen).

The Ti65 alloy contains phase-stabilizing elements, such as Si and Zr. During the high-temperature and long-duration thermal exposure, silicides will precipitate at the *α*/*β* phase interface. The silicides are divided into S1-type (TiZr)_5_Si_3_ and S2-type (TiZr)_6_Si_3_ [15], with the S1-type often appearing rod-shaped or cylindrical, while the S2-type is generally spherical or ellipsoidal [16]. Figure 3a–f, respectively, show the TEM images of the silicides in the Ti65 alloy before and after the thermal exposure at 450 °C, 500 °C, 550 °C, 600 °C, and 650 °C for 100 h. Ellipsoidal S2-type (TiZr)_6_Si_3_ silicides were observed at the *α*/*β* phase interface in the Ti65 alloy [15,16]. Based on numerous TEM images, the statistical results of the major and minor axes of the ellipsoidal silicides at the *α*/*β* phase interface in the Ti65 alloy before and after the thermal exposure are shown in Figure 3g and Figure 3h, respectively. With the increase in *T_e_*, both the major and minor axes of the ellipsoidal silicides in the Ti65 alloy increase.

**Figure 4:**
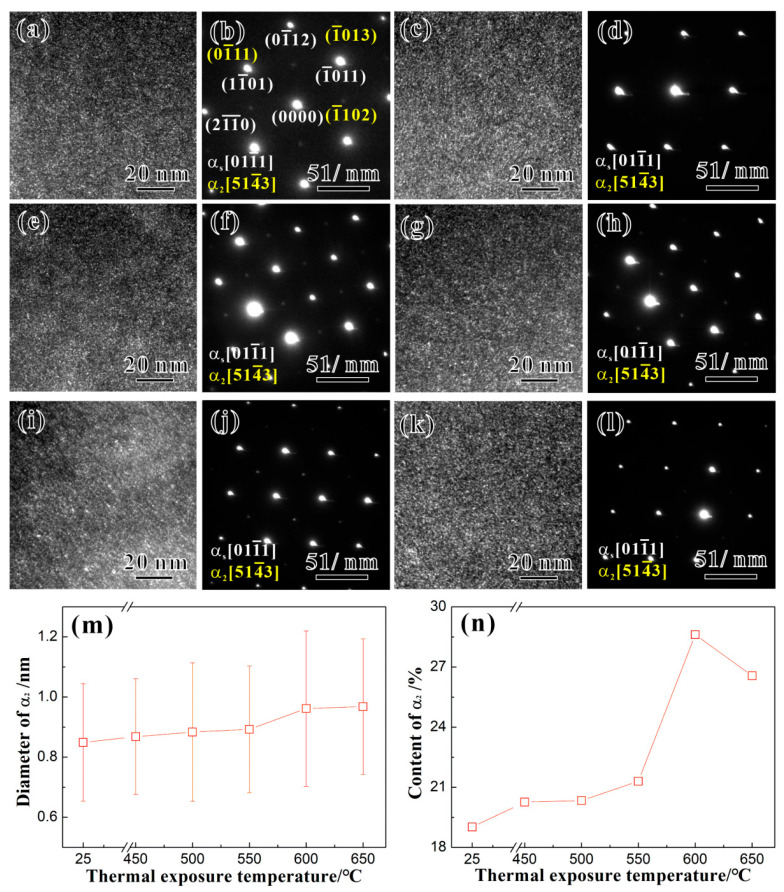
TEM images and corresponding SAED patterns of *α*_2_ phases in the Ti65 alloy (**a**,**b**) before and after the thermal exposure for 100 h at temperatures (HET) of (**c**,**d**) 450 °C, (**e**,**f**) 500 °C, (**g**,**h**) 550 °C, (**i**,**j**) 600 °C, and (**k**,**l**) 650 °C, respectively; and relations between the (**m**) diameter or (**n**) content of *α*_2_ phases with the heat exposure temperature (25 °C represents the unexposed specimen).

Figure 4a,c,e,g,i,k show the dark-field TEM images along the axis of the secondary lath *α_s_* phase along [01$\overset{-}{1}$1] in the Ti65 alloy before thermal exposure and after the thermal exposure at 450 °C, 500 °C, 550 °C, 600 °C, and 650 °C for 100 h. Figure 4b,d,f,h,j,l show the selected area electron diffraction (SAED) patterns corresponding to Figure 4a,c,e,g,i,k. Both before and after the thermal exposure, the Ti65 alloy contains a high-density and evenly distributed nanoscale spherical *α*_2_ ordered phase [17]; analysis and calibration of the superlattice structure of the diffraction spots in the SAED patterns reveal that the *α*_2_ phase is coherent with the matrix *α_s_* phase, and their crystallographic orientation relationship is [01$\overset{-}{1}$1]*α_s_*//[51$\overset{-}{4}$3]*α*_2_. Based on the TEM images, the statistical results of the diameter and content (area percentage) of the *α*_2_ phase within the secondary lath *α_s_* phase in the Ti65 alloy before and after the thermal exposure are shown in Figure 4m,n. Figure 4m shows that there is no significant change in the diameter of the *α*_2_ phase as *T_e_* < 500 °C compared to before thermal exposure (data corresponding to 25 °C). However, as *T_e_* ≥ 500 °C, the diameter of the *α*_2_ phase gradually increases with the increase in *T_e_*. As *T_e_* ≥ 600 °C, the increase in diameter becomes increasingly evident. Figure 4n shows that the content of the *α*_2_ phase increases initially and then decreases as *T*_e_ increases. As *T_e_* = 600 °C, the content of the *α*_2_ phase reaches its maximum.

### 3.2. Characterization of Oxide Layer

**Figure 5:**
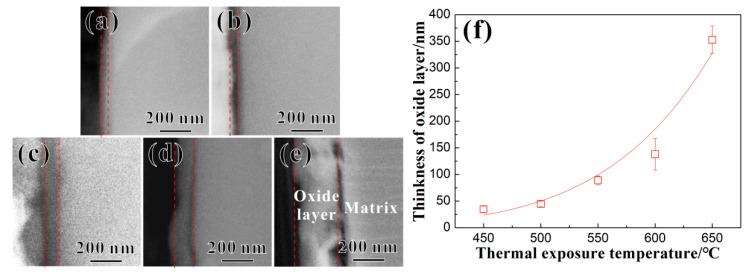
SEM images of Ti65 alloy with oxide layers after the thermal exposure for 100 h at temperatures of (**a**) 450 °C, (**b**) 500 °C, (**c**) 550 °C, (**d**) 600 °C, and (**e**) 650 °C, respectively; (**f**) statistics of thicknesses of oxide layers vs. thermal exposure temperatures (The red dashed lines indicate the oxide layer region in (**a**–**e**)).

Figure 5a–e show SEM images of the oxide layer of the Ti65 alloy after the thermal exposure at 450 °C, 500 °C, 550 °C, 600 °C, and 650 °C for 100 h, respectively. The red dashed lines indicate the oxide layer region. After the thermal exposure, there is a clear boundary between the oxide layer and the matrix of the Ti65 alloy, and the thickness of the oxide layer gradually increases with an increasing *T_e_*. Based on numerous SEM images, the statistical results of the oxide layer thickness after the thermal exposure in the Ti65 alloy are shown in Figure 5f. The oxide layer thickness shows an exponential growth trend with an increasing *T_e_*. However, overall, even after the thermal exposure at 650 °C for 100 h, the oxide layer thickness of the Ti65 alloy is only 353 nm, demonstrating good oxidation resistance.

**Figure 6:**
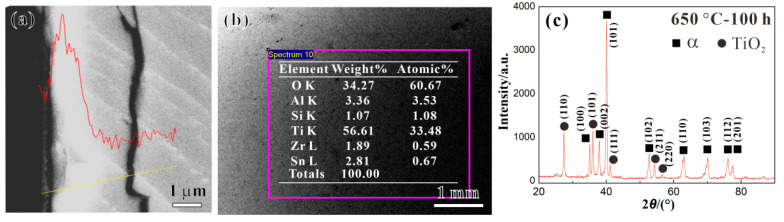
(**a**) Cross-section mapping, (**b**) surface mapping, and (**c**) XRD pattern of oxide layer of Ti65 alloy after the thermal exposure for 100 h at a temperature of 650 °C (The red line indicates the oxide content along the yellow line in (**a**)).

Figure 6a,b present the cross-section line scanning (along the yellow line) and surface map scanning (in the pink box area) of the oxide layer of the Ti65 alloy after the thermal exposure at 650 °C for 100 h, respectively. Firstly, it is evident from Figure 6a that the surface oxygen content of the Ti65 alloy is significantly higher compared to its internal composition. Moreover, there exists a gradual decrease in oxygen content from the surface towards the interior of the Ti65 alloy (indicated by the red line). Meanwhile, from Figure 6b, it can be seen that the main components of the oxide layer after the thermal exposure on the Ti65 alloy are Ti and O, accounting for over 90 wt% of the total elemental content, with an atomic ratio of Ti to O of around 2:1. Figure 6c presents the XRD analysis results of the oxide layer of the Ti65 alloy after the thermal exposure at 650 °C for 100 h. XRD results indicate that the diffraction peaks correspond mainly to the *α*-Ti matrix with (101), (100), and (002) crystal planes and the rutile structure TiO_2_ with (110), (101), and (211) crystal faces [18]. Therefore, it is suggested that the oxide layer of the Ti65 alloy after the thermal exposure mainly consists of a rutile structure TiO_2_.

### 3.3. Tensile Properties

**Figure 7:**
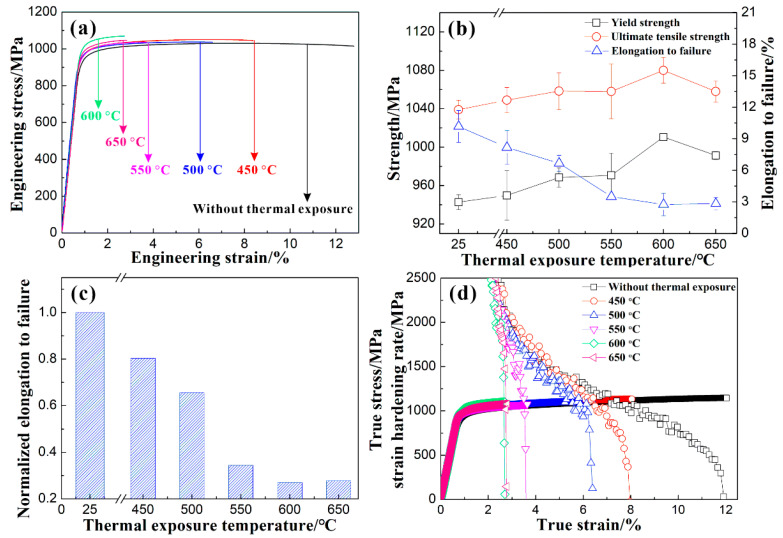
(**a**) Engineering stress–strain curves, (**b**) relations between strength or elongation to failure with thermal exposure temperature, (**c**) normalized elongation to failure as a function of thermal exposure temperature, and (**d**) true stress/strain hardening rate vs. true strain curves of Ti65 alloy without and with thermal exposing for 100 h at different temperatures (25 °C represents the unexposed specimen).

Figure 7a,b show the engineering stress–strain curves of the Ti65 alloy before and after the thermal exposure, as well as the variations in yield strength, ultimate tensile strength, and elongation at fracture. Overall, compared to the specimens before thermal exposure, the strength of the specimens exposed thermally at different temperatures for 100 h has increased, while the plasticity has decreased. The yield strength and ultimate tensile strength of the Ti65 alloy both show a trend of initially increasing and then decreasing with the increase in *T_e_*, reaching their maximum values after the thermal exposure at 600 °C for 100 h. On the other hand, the elongation at fracture of the Ti65 alloy decreases gradually with the increase in *T_e_*. In order to determine the room temperature tensile plasticity reduction of the Ti65 alloy after the thermal exposure, the elongation to failure of the Ti65 alloy after the thermal exposure at 450~650 °C for 100 h is normalized by the elongation to failure of the unexposed Ti65 alloy, and the results are shown in Figure 7c. The results indicate that at *T_e_* below 550 °C, the plasticity of the Ti65 alloy exhibits a slight decrease, yet still remains above 65% compared to the unexposed specimen. However, at *T_e_* exceeding 550 °C, the plasticity of the Ti65 alloy experiences a significant reduction and only reaches approximately 30% of the unexposed specimen. Figure 7d presents the variation in the true stress and strain hardening rate as a function of the true strain of the Ti65 alloy before and after the thermal exposure. As observed in Figure 7d, the strain hardening rate of the unexposed specimens gradually decreases with an increasing true strain, and even after plastic instability occurs in the alloy, it retains a certain degree of non-uniform plastic deformation capability. In comparison, for specimens exposed to temperatures below 500 °C, the strain hardening rate continues to decrease gradually with an increasing true strain. However, as the specimen undergoes plastic instability, the non-uniform plastic deformation capability decreases with the increase in *T_e_*. For specimens exposed to temperatures above 550 °C, the strain hardening rate decreases sharply with an increasing true strain, without showing a distinct stage of uniform deformation. This indicates that the alloy fractures prematurely before experiencing uniform deformation, suggesting that this premature failure may be related to the early cracking of the oxide layer on the specimen’s surface and the oxygen diffusion zone introducing a notch effect leading to premature failure of the matrix.

### 3.4. Tensile Fracture Behavior

**Figure 8:**
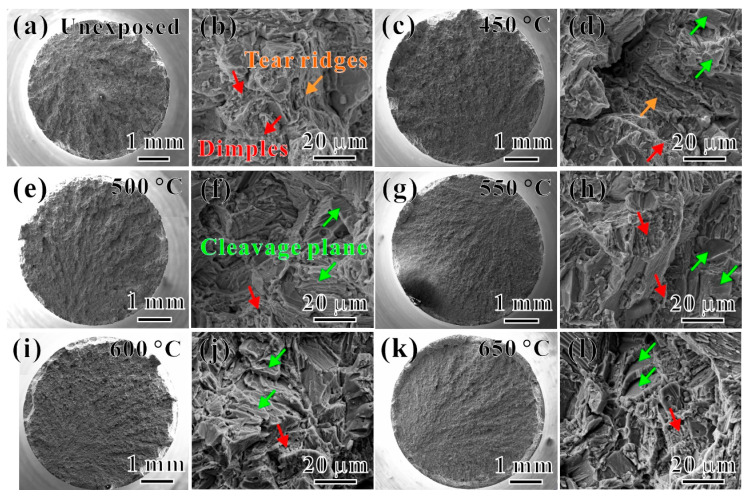
SEM images of tensile fractured surfaces of Ti65 alloy (**a**,**b**) without the thermal exposure and after the thermal exposure for 100 h at temperatures of (**c**,**d**) 450 °C, (**e**,**f**) 500 °C, (**g**,**h**) 550 °C, (**i**,**j**) 600 °C, and (**k**,**l**) 650 °C, respectively (orange arrows, tear ridges; red arrows, dimples; and green arrows, cleavage plane).

Figure 8a,b show the macroscopic and microscopic SEM images of the tensile fracture surface of the Ti65 alloy before the thermal exposure, respectively. One can find that the macroscopic fracture surface of the Ti65 alloy before thermal exposure exhibits a rough “dark gray” fibrous fracture surface, with shear lips oriented at approximately 45° to the tensile axis (Figure 8a). The fracture surface displays clear tear ridges and dimples of the ductile fracture, as indicated by the red arrows in Figure 8b. Figure 8c–l show the macroscopic and microscopic SEM images of the tensile fracture surfaces of the Ti65 alloy after the thermal exposure at 450~650 °C for 100 h. By comparing the macroscopic fracture morphology, one can see that as *T_e_* increases, the fracture morphology transits from a rough uneven state to a flat state, indicating a decrease in the plasticity of the specimen with an increasing *T_e_*. Furthermore, compared with the microscopic morphology of the fracture surfaces, numerous elongated fracture planes and bright tear ridges are observed on the fracture surface of all specimens after the thermal exposure at various temperatures, corresponding to the lamellar *α_s_* phases in the microstructure, indicating that the fracture in some regions of the specimen occurs along the lamellar *α_s_* phases. When *T_e_* is relatively low (Figure 8d,f corresponding to thermal exposures at 450 °C and 500 °C, respectively), in addition to observing the aforementioned fracture planes, tear ridges, and localized dimples on the microscopic fracture surface, brittle cleavage features are already visible on the fracture surface (as indicated by the blue arrows in the images). Referring to the changes in tensile properties shown in Figure 7, it can be inferred that although the plasticity of the specimen is decreasing, it still exhibits “ductile fracture” characteristics. As *T_e_* increases from 550 °C to 650 °C (Figure 8g,h,l), more and more cleavage planes and cleavage steps appeared on the tensile fracture surface (as indicated by the blue arrows), indicating a transition of the tensile fracture mode from ductile fracture to cleavage fracture, representing a mixed fracture mode of the ductile and cleavage fracture.

**Figure 9:**
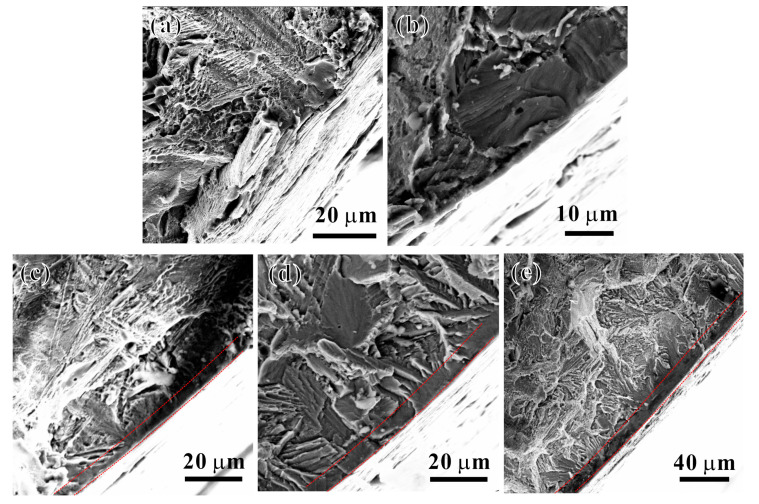
SEM images of tensile fractured surfaces at edge zones of samples after the thermal exposure at temperatures of (**a**) 450 °C, (**b**) 500 °C, (**c**) 550 °C, (**d**) 600 °C, and (**e**) 650 °C, respectively (Red dashed lines indicate the intergranular brittle fracture regions).

Figure 9 shows the SEM observations of the tensile fracture edge of the specimens after the thermal exposure at different *T_e_*s for 100 h. The edge regions of the fracture exhibit a brittle oxidation layer and an oxygen diffusion zone. During the tensile loading process, the surface tends to crack first, forming intergranular brittle fracture regions (as indicated by the red dashed line in the image) [19]. The tensile fracture surfaces of the specimens exposed at *T_e_* = 550 °C and above are shown in Figure 9c–e; it is evident that clearly defined and continuous intergranular brittle fracture regions are present and increase gradually with the increase in *T_e_*. Additionally, many cleavage planes and river-like patterns of brittle macroscopic fracture characteristics are observed near the intergranular brittle fracture zones. This suggests that the formation of the oxidation layer is an important factor contributing to the decreased plasticity of the Ti65 alloy after thermal exposure.

## 4. Discussion

### 4.1. Influence of Thermal Exposure Temperature on Microstructures

The above results indicate that both before and after the thermal exposure, S2-type silicides exist at the *α*/*β* phase boundaries in the Ti65 alloy, and their size increases with an increasing *T_e_*. For the Ti65 alloy, the Si element has a low solubility limit, so during aging treatments or prolonged high-temperature thermal exposure, silicides precipitate in the *β* phase near the *α*/*β* phase boundary [20]. The precipitation and growth of silicides in the aforementioned processes are mainly controlled by the diffusion of elements, such as Si and Zr that form the *β* phase in the alloy, and the growth rate is proportional to the content of these elements [20]. After the thermal exposure at different *T_e_*s, the Ti65 alloy shows a similar content of the *α_p_* phase, resulting in a comparable number of *α*/*β* phase boundaries. However, with an increasing *T_e_*, the accumulation of elements, such as Si and Zr, at the phase boundaries increases, leading to a continuous rise in the element content near the *α*/*β* phase boundaries. Consequently, the Ti65 alloy thermally exposed at 650 °C for 100 h exhibits the largest size of silicides.

The results also reveal that both before and after the thermal exposure, the Ti65 alloy contains high-density and uniformly distributed spherical nanoscale *α*_2_ phases, and the size of the *α*_2_ phases remains initially unchanged and slightly increases later as the *T_e_* increases, while the content shows an increasing trend followed by a decrease. In titanium alloys, the growth of *α*_2_ phases is mainly controlled by the diffusion of Al elements, and the growth rate is positively correlated with the Al content in the *α* phase. During the prolonged thermal exposure, Al elements continuously accumulate in the *α* phase [21], and the higher the *T_e_*, the higher the accumulation of Al in the *α* phase. For most titanium alloys, the precipitation temperature range of *α*_2_ phases is 500 °C to 760 °C, with the maximum precipitation rate at 600 °C. Therefore, when *T_e_* < 500 °C (outside the precipitation temperature range of *α*_2_ phases), the size of the *α*_2_ phases does not show significant changes. However, as *T_e_* > 500 °C, the size of the *α*_2_ phases gradually increases with an increasing *T_e_*. Additionally, as shown in Figure 4n, the content of *α*_2_ phases initially increases with an increasing *T_e_*, reaching a peak at 600 °C. This indicates that *α*_2_ phases in the Ti65 alloy have the highest precipitation rate at 600 °C. When *T_e_* = 650 °C, the precipitation rate decreases and the *α*_2_ phase content diminishes [22].

### 4.2. Effect of Thermal Exposure on the Strength of the Alloy

The comparison in Figure 2 indicates that there is no significant change in the microstructure of the Ti65 alloy matrix before and after the thermal exposure. Both instances show a dual-phase microstructure composed of equiaxed primary *α* phases and *β_trans_* phases, with similar contents of *α_p_* phases and *α_s_* phase layer widths. Therefore, the change in the tensile properties of the Ti65 alloy is mainly influenced by the oxide layer generated during the prolonged high-temperature thermal exposure and the precipitation phases within the matrix. For alloys with a brittle fracture zone on the surface, the strength can be expressed based on the rule of mixtures as follows:

*σ*_*s*_ = *σ*_*b*_*f*_*b*_ + *σ*_0_*f*_0_ (1)

Here, *σ_b_* and *f_b_* are the tensile strength and volume fraction of the matrix, and *σ*_0_ and *f*_0_ are the tensile strength and volume fraction of the brittle fracture zone, respectively. From the experimental results, the oxide layer thickness is on a nanometer scale, while the diameter of the thermal exposed tensile specimen is 5 mm. Therefore, the *f*_0_ value is much smaller than the *f_b_* one, so the contribution of the brittle oxide layer to strength can be neglected.

After the prolonged high-temperature thermal exposure, the alloy will precipitate *α*_2_ phases and S2-type silicides within the matrix. The *α*_2_ phase with a coherency relationship with the matrix effectively enhances the strength through interactions with dislocations. Zhang et al. [23] have shown that when the *α*_2_ phase size is less than 6 nm, dislocations will cut through the *α*_2_ phase. Here, the *α*_2_ phase size inside the Ti65 alloy after the thermal exposure is around 0.8 nm; thus, the cutting mechanism should be activated as mobile dislocations interact with tiny *α*_2_ phases. Such strengthening effects may be mainly contributed by coherency strengthening, modulus strengthening, chemical strengthening, and order strengthening, and the shear stress increment can be evaluated by [24] the following:

Δ*τ_coh_* = 7|*ε_coh_*|^3/2^ *G* (*rf*/*b*)^1/2^ (2)

Δ*τ_GP_* = 0.01 *ε_G_*^3/2^ *G* (*rf*/*b*)^1/2^ (3)

Δ*τ_chem_* = 2 *G* (*γ_s_*/*Gr*)^3/2^ (*rf*/*b*)^1/2^ (4)

**Figure 10:**
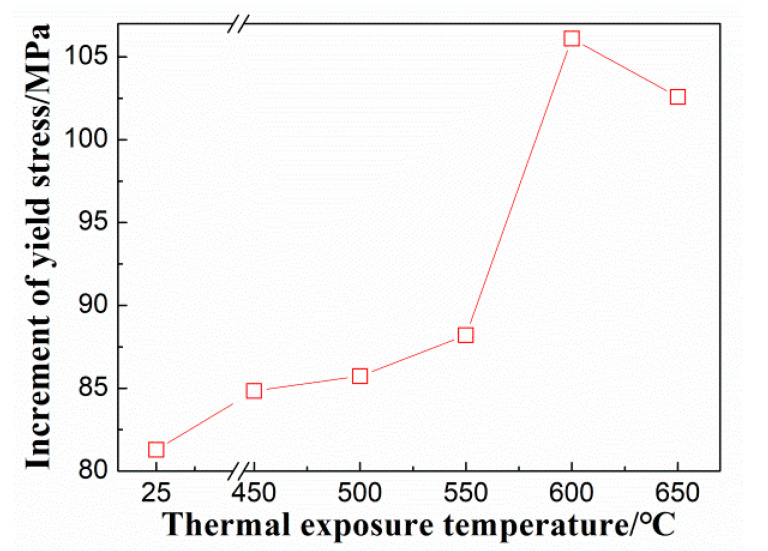
Calculation increments of shear stress caused by order strengthening of *α*_2_ phases at different heat exposure temperatures for 100 h (25 °C represents the unexposed specimen).

Δ*τ_tod_* = 0.7 *G* (*γ_APBE_*/*Gb*)^3/2^ (*rf*/*b*)^1/2^ (5)

where *r* and *f* represent the radius and volume fraction of the *α*_2_ phase, respectively. *G* and *b* are the shear modulus and Burgers vector of dislocations, respectively. *ε_coh_* and *ε_G_* signify the lattice misfit and the shear modulus mismatch between the matrix and the *α*_2_ phase, respectively. *γ_s_* indicates the interfacial energy between the *α*_2_ phase and the matrix, and *γ_APBE_* denotes the anti-phase boundary energy (APBE) formed by dislocations shearing through the *α*_2_ phase. From Equations (2)–(5), one can find that the four strengthening effects mentioned above have a square root relationship with r and f of the *α*_2_ phase, indicating that the precipitation and growth of the *α*_2_ phase will enhance the strength. On the other hand, the *α*_2_ phase in the Ti65 alloy is a long-range ordered phase. When dislocations shear through the *α*_2_ phase, a larger APBE will be generated. Therefore, the increase in APBE in the Ti65 alloy is the main cause of strengthening, and the increment in strength caused by this strengthening effect can be calculated using Equation (5) [24]. Given that *G* = 43.6 GPa [25] and *b* = 0.259 nm. *γ_APBE_* in the alloy is influenced by the Ti/Al ratio [26], with Ti/Al taken as 15, and the *γ_APBE_* value formed by dislocation shearing through the *α*_2_ phase is taken as 320 mJ/m^2^. The variation in the increment of the shear stress caused by the ordered strengthening in the alloy at different *T_e_*s is calculated and presented in Figure 10. It is clear that the change in the shear stress increment due to the ordered strengthening by the *α*_2_ phase at different *T_e_*s is consistent with the trends in the yield strength of the Ti65 alloy at various *T_e_*s. This analysis indicates that the mechanical properties of the alloy after the thermal exposure are mainly controlled by the precipitation of the *α*_2_ phase within the alloy during the thermal exposure process. Overall, the tensile strength of the Ti65 alloy is mainly dominated by the stability of the microstructure during the thermal exposure process. The mechanical performance of the alloy after the thermal exposure is primarily controlled by the precipitation of *α*_2_ phases within the alloy during the thermal exposure process.

### 4.3. Influence of Thermal Exposure on Plasticity

**Figure 11:**
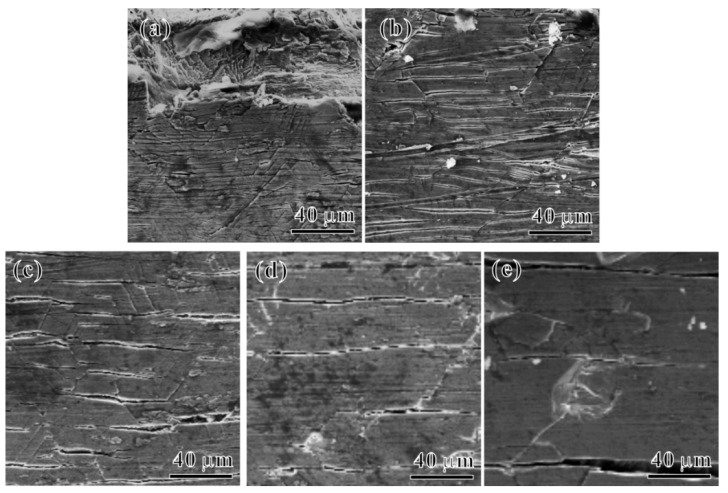
SEM images of surface tensile deformation of samples with the oxide layer under thermal exposure temperatures at (**a**) 450 °C, (**b**) 500 °C, (**c**) 550 °C, (**d**) 600 °C and (**e**) 650 °C, respectively (loading direction is vertical).

Figure 11 shows SEM images of the surface morphology of tensile specimens of the Ti65 alloy after 100 h of thermal exposure at different *T_e_*s. Significant cracking behaviors are visible on the specimen surfaces after tensile deformation following thermal exposure at various *T_e_*s. Characterization of the tensile fracture surfaces (Figure 9) reveals that after the prolonged high-temperature thermal exposure, a brittle layer composed of an oxide layer and an oxygen diffusion layer formed on the surface region of the Ti65 alloy. This results in the formation of cracks on the specimen surface under tensile loading, with preferential cracking. These cracks propagate from the surface towards the interior, leading to the appearance of notches in the alloy matrix, and stress concentration occurs at the crack tip. When the stress at the notch tip exceeds the yield strength, the crack propagates into the matrix, causing premature failure fracture of the specimen and ultimately deteriorating the tensile plasticity of the alloy [19,27]. With an increase in *T_e_*, the thicknesses of the oxide layer and oxygen diffusion layer of the Ti65 alloy gradually increase, leading to a more pronounced decrease in the plasticity of the alloy.

**Figure 12:**
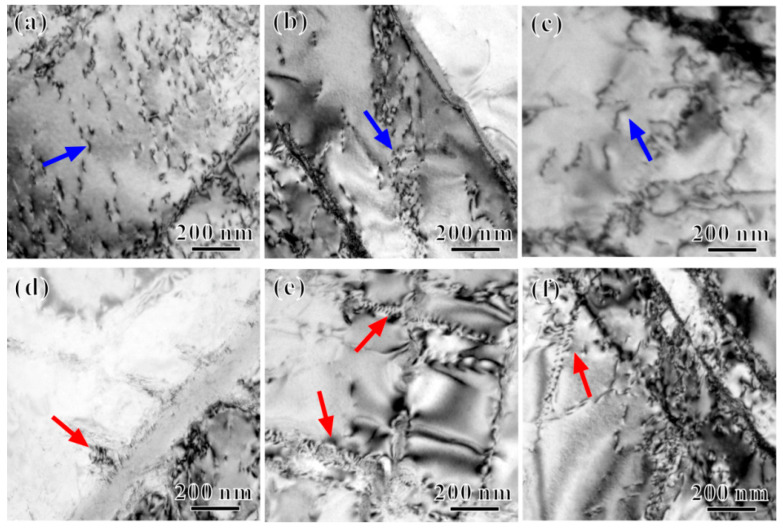
Dislocation behavior of tensile fractured specimens (**a**,**d**) before and after the thermal exposure for 100 h at temperatures of (**b**,**e**) 600 °C and (**c**,**f**) 650 °C, respectively (Blue and red arrows indicate the dislocation glide and dislocation accumulations characteristics, respectively).

To examine the influence of the precipitates on plasticity, Figure 12 shows TEM images of the dislocation configurations observed in the Ti65 alloy specimens before and after the thermal exposure. Figure 12a–c show some dislocation glide characteristics within the lamellar *α*_2_ phase in samples before thermal exposure and after exposure to 600 °C and 650 °C, indicated by blue arrows. With an increasing *T_e_*, the dislocation glide characteristics become more pronounced. Although the interaction between the *α*_2_ phase and mobile dislocations can significantly enhance the strength of the alloy, the shearing of small, ordered *α*_2_ phases mediated by dislocations may promote the formation of slip bands within the alloy. This process leads to strain localization and crack nucleation, with cracks rapidly propagating along the slip bands, ultimately reducing the alloy’s plasticity [28,29]. With an increasing *T_e_*, both the size and content of the *α*_2_ phase within the alloy increase (slightly decreasing at 650 °C due to limited resorption), exacerbating strain localization induced by dislocation glide and further reducing plasticity. Consequently, influenced by the size and content of the *α*_2_ phase, the elongation to fracture of the Ti65 alloy subjected to thermal exposure at *T_e_* = 600 °C reaches its lowest value.

Additionally, as shown in Figure 12d–f, there are evident dislocation accumulations at the silicides along the *α*/*β* phase boundary. During tensile deformation, as the strain increases, mobile dislocations shearing through the *α*_2_ phase in the matrix continue to glide and encounter the silicides, resulting in their accumulation at the continuous *α*/*β* phase boundary. The larger the silicide particles, the greater the stress concentration at the *α*/*β* phase boundary, which can lead to early crack nucleation and the degradation of plasticity [10,12]. Thus, the decrease in the plasticity of the Ti65 alloy due to the presence of silicides and the *α*_2_ phase may have an additive effect. As the *T_e_* increases, the size of the S2-type silicides also increases, leading to the more significant accumulation of dislocations near them and consequently a more pronounced reduction in plasticity. Overall, the tensile plasticity of the Ti65 alloy is primarily influenced by the combined effects of surface stability and microstructural stability during the thermal exposure process.

## 5. Conclusions

(1) After 100 h of thermal exposure in the air at 450~650 °C, the size and content of the *α*_2_ phase and silicides in the Ti65 alloy increase with an increasing thermal exposure temperature. Simultaneously, a TiO_2_ oxide layer forms on the alloy surface, with the thickness exponentially growing as the thermal exposure temperature increases.

(2) The strength of the alloy increases after the thermal exposure compared to that before thermal exposure, while the plasticity exhibits an opposite trend. With an increasing thermal exposure temperature, the strength of the alloy initially increases but then decreases, while the plasticity continues to decrease. After 100 h of thermal exposure at 600 °C, the yield strength and tensile strength of the alloy are at their highest, measuring 1010 ± 3.54 MPa and 1080 ± 13.43 MPa, respectively, with the lowest elongation after fracture at 2.75 ± 0.58%.

(3) The increase in the thickness of the surface TiO_2_ oxide layer after the prolonged thermal exposure leads to premature failure and thus decreased plasticity. Additionally, the *α*_2_ phase within the matrix promotes dislocation glide, while the silicides at the *α*/*β* phase boundary hinder dislocation movement, causing dislocation accumulations, both of which promote crack nucleation and degrade the plasticity.

## Figures and Tables

**Table 1:** Chemical composition of Ti65 alloy (wt%).

Element	Al	Sn	Zr	Mo	Si	Nb	Ta	W	C	Ti
wt%	5.75	4.00	3.50	0.50	0.40	0.30	1.00	0.80	0.05	Bal.

## Data Availability

The original contributions presented in the study are included in the article; further inquiries can be directed to the corresponding authors.
